# Adjuvant treatment of high-risk melanoma – cost-effectiveness analysis of treatment options for BRAF 600 mutated tumors

**DOI:** 10.1186/s13561-021-00347-7

**Published:** 2022-01-20

**Authors:** Steffen Wahler, Alfred Müller, Sabine Fuchs, Johann-Matthias von der Schulenburg

**Affiliations:** 1 St. Bernward GmbH, Friedrich-Kirsten-Straße 40, D-22391 Hamburg, Germany; 2 Analytic Services GmbH, Jahnstr. 34c, D-80469 Munich, Germany; 3 Novartis Pharma GmbH, Roonstr. 25, D-90429 Nuremberg, Germany; 4 Universität Hannover, Welfengarten 1, D-30167 Hannover, Germany

**Keywords:** Melanoma, Cost-effectiveness, Checkpoint-inhibition, Targeted therapy, Adjuvant treatment

## Abstract

**Introduction:**

Until recently, adjuvant treatment options for higher stage resectable cutaneous melanoma were limited. Two studies with a similar set-up, published 2017, led to registration of targeted therapy for BRAF-mutated melanoma with dabrafenib and trametinib as well as of the immunotherapy with nivolumab irrespective of BRAF-mutation status. Both options have been positively assessed in Germany since 2019 for the adjuvant treatment of BRAF-V600 mutated melanoma. This study evaluates the cost-effectiveness of both treatment alternatives (dabrafenib/trametinib and nivolumab) against observation as a comparative therapy from the perspective of German statutory health funds.

**Methods:**

Partitioned survival analysis based on published survival curves for the investigated treatment options was used for a cohort model for the health states relapse free survival, progression, and death. The partitioned survival analysis approach was based on the survival curves published for the key studies Combi AD and Checkmate-238. The modelling was performed for the remaining lifetime for a cohort with starting age of 50 years. For extrapolation of the survival curves, convergence to general population mortality rates was assumed in the long term. Within the progression state, a Markov model uses three levels of progressions (locoregional, distant metastases with 1st and 2nd line treatment). Lifetime treatment costs were calculated using the German statutory health fund reimbursement scheme. Quality adjusted life years (QALYs) associated to the health states were adopted from previously published utilities based on the Combi AD study.

**Results:**

The treatment with dabrafenib/trametinib yielded an increase in quality adjusted life years of 2.28 QALY at an incremental lifetime cost of 86.1 T€. The incremental cost effectiveness ratio of dabrafenib/trametinib and nivolumab was comparable with 37.8 T€/QALY and 30.0 T€/QALY, respectively. Several sensitivity analyses proved the result to be insensitive. General model parameters like discount rate and length of the time horizon had stronger influence. For nivolumab, the model showed lower discounted lifetime costs (118.1 T€) compared to dabrafenib/trametinib [155.1 T€], associated with a lower gain in QALYs (1.64 years) compared to observation.

**Conclusion:**

Both dabrafenib/trametinib and nivolumab turned out to be cost effective within internationally accepted Incremental Cost Effectiveness Ratio (ICER) thresholds with comparable cost effectiveness ratios.

## Background

Melanoma is a cancer that develops from melanocytes and is typically located in parts of the body that have been overexposed to the sun [1]. Global incidence for cutaneous melanoma is increasing and was estimated 288,000 patients in 2018 [2–4], resulting in around 55,000 deaths annually [5]. In Germany in 2016 the incidence was approximately 23,000 patients [6, 7].

Localized melanoma is usually surgically resected. This regularly cures stage I and II disease [8]. Higher stage disease has an elevated risk of recurrence. For stage IIIA, IIIB, and IIIC disease five years data showed relapse in 37, 68, and 89% of resected patients [7, 8] and 5-year survival rates from time of first relapse of 20, 20, and 11% [9]. Thus, Nading indicated in 2009 that more than half of patients in stage III died within ten years after first diagnosis [8, 10].

In the past years, adjuvant treatment options for patients with resected melanoma with high risk of relapse have been constrained [11]. Different therapies had been explored but did not lead to improved overall survival [12–14]. Interferon alpha-2b was a first registered option for that indication, but with limited survival benefit and an unfavorable side effect profile [11, 15–17].

In the last decade, targeted therapy and immunotherapy several became new therapeutic options, for advanced stage melanoma. They demonstrated efficacy and improved the outcome for melanoma patients [18, 19]. Ipilimumab, an anti-CTLA-4 antibody, was the first new drug which significantly improved overall survival (OS) versus placebo [20, 21]. Further CTLA-4 and PD-1 immune-checkpoint inhibitors followed in demonstrating that immunotherapy improves survival for defined patient cohorts [22, 23].

The success of kinase inhibitors as targeted therapy was triggered by the detection of activating somatic BRAF V600 mutations in melanoma cells [23]. Those are found in around 45% of advanced melanomas and result in consecutive activation of the MAPK (Mitogen Activated Protein Kinase) pathway [24, 25]. The blocking of this MAPK pathway activation by a combination of BRAF inhibitors and MEK inhibitors demonstrated significant clinical benefit in patients with BRAF V600-mutated melanomas [26–29].

First trials with the new therapeutic options could prove enhanced relapse-free survival [30, 31]. Almost simultaneously two new treatments, the targeted therapy of combined dabrafenib and trametinib, and the immunotherapy with checkpoint-inhibitor nivolumab, underwent two major phase-III trials in comparable populations with advanced cutaneous melanoma for adjuvant therapy after resection. Both trials, COMBI AD [32] for the targeted combination and CheckMate 238 [33] for the checkpoint-inhibitor were base for registrations by EMA and FDA [34–36]. They were published back-to-back in 2017 in the same journal. Both treatments demonstrated significantly improved outcomes thus far in relapse free survival and distant metastasis free survival. COMBI AD was exclusively for patients with a proven BRAF-mutation.

The follow-up periods for the registration trials mentioned above (Combi-AD, Checkmate-238) have not yet finished. The latest follow-up for Combi-AD was a publication of DMFS (Distant Metastasis Free Survival), RFS (Relapse Free Survival) and OS (Overall survival) at 60 months. Updates for Checkmate-238 OS, DMFS and RFS at 48 months were recently published [37].

Since the treatment options under consideration are new and the associated randomized studies are still in the follow-up stage, there are few published cost-effectiveness studies on the use of immunosuppressants in the adjuvant therapy of melanoma. Until 2010, cost-effectiveness studies concentrated on the use of high-dose interferon (see, e.g. [38, 39]). Since 2019, cost-effectiveness analyses of Gerbasi et.al [40]. (combination therapy dabrafenib/trametinib vs. observation), Bensimon et.al [41]. (pembrolizumab vs. observation), Salans et.al [42]. (ipilimumab vs. high-dose interferon), and Gao et.al [43]. (combination therapy dabrafenib/trametinib vs. vemurafenib) have been published.

In Germany, the reimbursement process usually contains only a benefit assessment. An empirical study [44] showed, however, that both the negotiated annual treatment costs of comparator drugs and the added benefit have a significant effect on the actual negotiated drug price. The present study is adding the evaluation of the cost-effectiveness of the treatment alternatives (dabrafenib/trametinib and nivolumab) against observation as a comparative therapy from the perspective of German Statutory Health Insurance (SHI) funds.

## Methods

The present study compares treatment alternatives for patients with resected BRAF V600 mutant stage III melanoma. It focuses on a cost-effectiveness comparison of the combination therapy dabrafenib/ trametinib (Tafinlar/Mekinist®, Novartis) with observation (routine surveillance). In addition, the treatment alternative nivolumab (Opdivo®, Bristol-Myers-Squibb) is compared with observation using the same model structure.

### Model setup

**Fig. 1:**
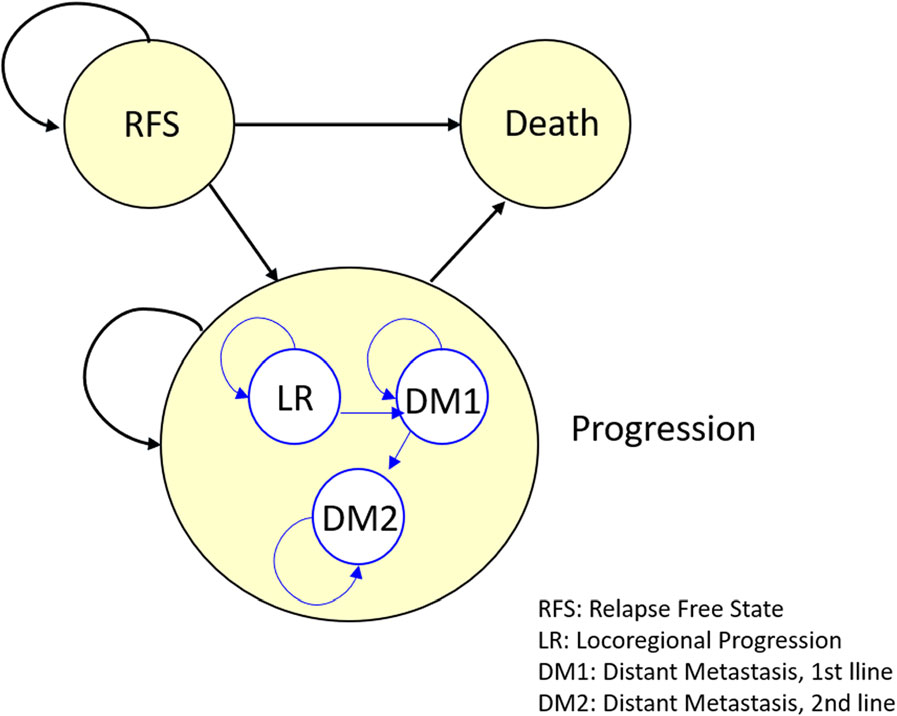
Model states and Markov sub model

The study uses partitioned survival analysis (Partitioned SA) as the primary modelling approach [45] with three states (relapse free survival, survival after progression, death). The proportion of participants within each state at a certain point in time is determined by the underlying survival curves. Within the progression state, a Markov sub-model was constructed to represent different states of progression (LR – locoregional progression, DM1 – distant metastasis, 1st line treatment, and DM2 – distant metastasis, 2nd line treatment (Fig. 1). The Partitioned SA model is a cohort model.

**Fig. 2:**
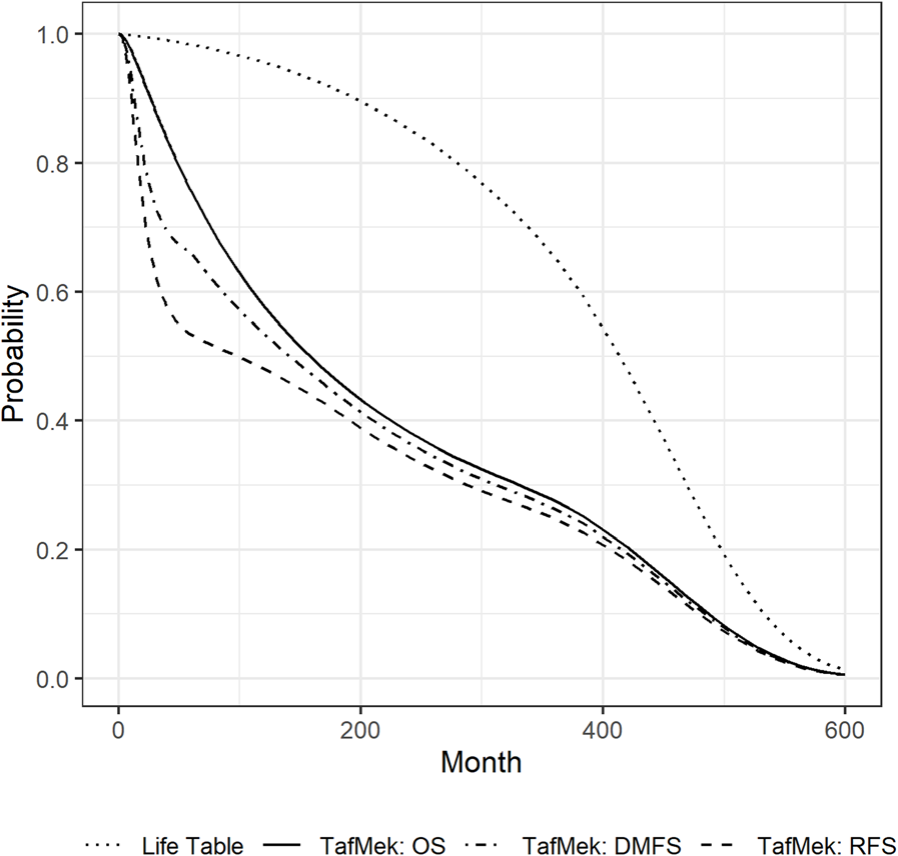
Survival curves related to combination therapy dabrafenib/trametinib. TafMek: dabrafenib/trametinib; RFS: relapse free survival; OS: overall survival; DM: distant metastases free survival

Long et.al [32]. published OS and DMFS data for dabrafenib/trametinib vs. Observation for the Combi AD study at 54 months of follow-up ([32], supplement). Hauschild et.al [46]. published corresponding RFS data at 60 months of follow-up. Patient level data were not available for any of the studies mentioned above. Published survival curves were digitized and converted into pseudo-event and censorship data applying the method published by Hoyle and Henley [47]. The resulting pseudo-event and censorship data were used to fit parametric survival models. Time-varying transition probabilities required for the Markov sub model were derived from the 1LPFS (1st line progression free survival) and 2LOS (2nd line overall survival) curves published by Gerbasi et.al. ([40], Fig. 2B and C).

Concerning background mortality and treatment costs, the model assumptions reflect a German setting assuming the cost-effectiveness perspective of the German Statutory Health Insurance Scheme. In the base case, the remaining lifetime of a cohort of 50-year-old patients is modeled with a cycle time of 6 months.

Lifetime treatment costs starting with the adjuvant treatment after the complete resection of the tumor affected region are the cost endpoint of the model. QALY after treatment start and remaining lifetime are primary resp. secondary effectiveness endpoints. The ICER of dabrafenib/trametinib vs. observation is the cost effectiveness endpoint of the model. Cost and effectiveness endpoints are discounted by 3% annually.

The lifetime horizon of the model requires the extrapolation of the survival curves beyond the limit determined by the follow-up periods of the underlying studies. In the short term (up to 60 months), the empirical Kaplan-Meier curves were fitted by parametric models. Log-logistic, lognormal, Weibull, gamma, and exponential were considered as candidates for the parametric hazard functions. Log-logistic (for RFS, DMFS) and lognormal model representations were selected based on the Akaike information criterion [48]. After the end of the follow-up period, a transitional period has been defined during which mortality rates converge towards the mortality of the general population. After this period, general mortality data using German life tables [49] are applied. For DMFS and RFS, survival curve extrapolations were defined in a similar way with event probabilities converging to the mortality of the general population.

### Projection of nivolumab results

For nivolumab vs. observation OS, RFS, and DMFS, recently published 4-year follow-up results for study Checkmate-238 [37] were adopted. A Bucher [50] indirect comparison related these results to the results of EORTC-18071 (ipilimumab vs. placebo [18]) using ipilimumab as bridge comparator following the technique outlined by Hemstock et.al [51]. The hazard ratio (HR) estimate for overall survival of nivolumab vs. observation was 0.635 (95% CI: 0.453–0.889). For relapse free survival, the hazard ratio estimate was 0.533 (95% CI: 0.417–0.681). The hazard ratio for distant-metastasis free survival was estimated at 0.600 (95% CI: 0.452–0.797). The results published by [51] were based on a previous data cut of Checkmate-238 and therefore did not include hazard ratios for overall survival. Their corresponding results were for the respective ITT populations were hazard ratio estimates of 0.53 (CI: 0.41–0.68) for RFS and 0.59 (CI: 0.44–0.78) for DMFS. Patient characteristics of Checkmate-238 and EORTC-071 studies were assessed by [51] and found to be balanced.

**Fig. 3:**
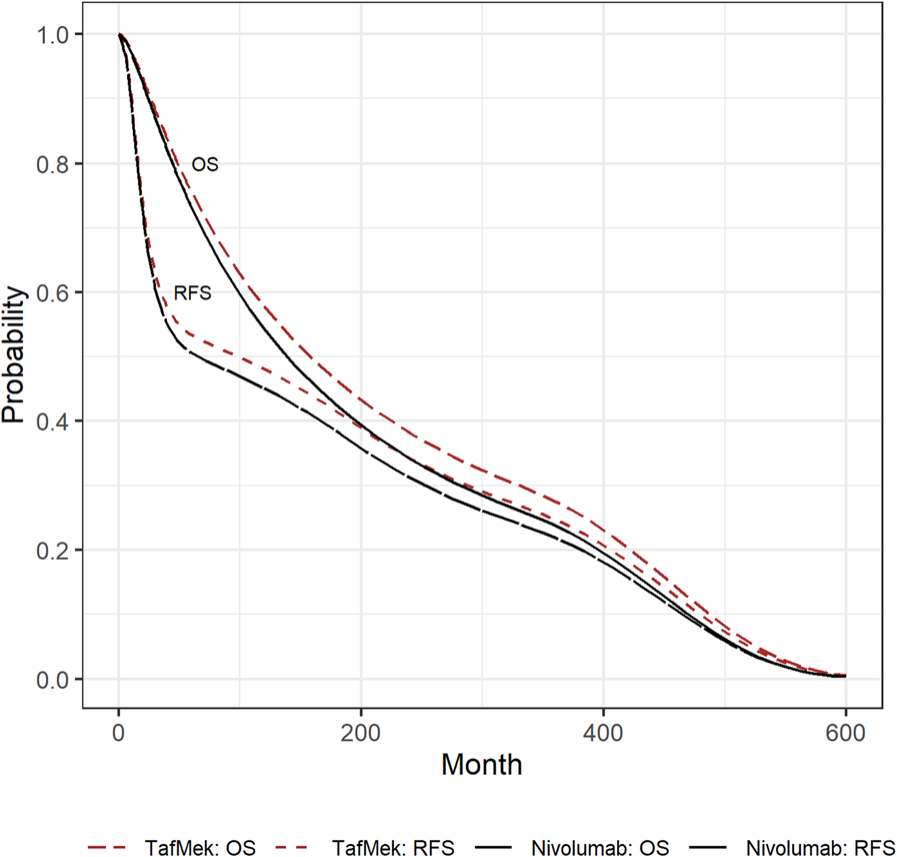
Survival curves combination dabrafenib/trametinib vs. Nivolumab. TafMek: dabrafenib/trametinib; Nivo: nivolumab; RFS: risk free survival; OS: overall survival

A second Bucher indirect comparison related these results to the results of Combi-AD (dabrafenib/ trametinib vs. observation [32, 46]) using observation as bridge comparator. The hazard ratio estimate for overall survival of dabrafenib vs. nivolumab with observation as bridge comparator was 0.8975 (95% CI: 0.575–1.400). For RFS and DMFS, the hazard ratio estimates were 0.9202 (95% CI: 0.673–1.259) and 0.8827 (95% CI: 0.612–1.274), respectively. The hazard ratio estimates were applied to the respective survival curves of dabrafenib/trametinib assuming proportional hazards over time (see Fig. 3).

Table 4 (appendix) provides an overview of the population characteristics of the three studies involved. There are differences in the inclusion of different stages of melanoma. In terms of demographics, the differences in the proportion of gender categories seem to be unbalanced, while age seems to be well balanced. The impact of the lack of balance can be verified by sensitivity analyses [51]. However, the corresponding analyses require the availability of patient level data, which was not the case here. Although the approach outlined above serves the goal of showing dabrafenib/trametinib and nivolumab in a common modeling framework, several methodological reservations remain. Details are discussed in the limitations section.

### Model parametrization

Within the Partitioned SA model setup, time-varying transition probabilities are defined by the underlying survival curves. Transition probabilities from the outer Partitioned SA model to the Markov progression sub model and within the Markov sub model require additional parameters. Transitions from the relapse free state to the progression state can be triggered by locoregional recurrences as well as by distant metastases. Following ([46], Table 1) It is assumed that 33% of the transitions are locoregional recurrences and the remaining 67% are distant metastases. Transitions from the locoregional state to 1st line distant metastasis treatment are informed by the DMFS survival curve with the additional assumption, that the hazard of progression into a distant metastasis state is increased by a factor 1.5 within the cohort of patients with locoregional progressions. Transitions from 1st line treatment to 2nd line treatment are informed by the 1LPFS survival curve ([40], Fig. 2B). Transitions from the distant metastases state to death are informed by the 2LOS survival curve ([40], Fig. 2C). Mortality in the loco-regional progression state is assumed to be lower compared to the distant metastasis states. Few publications investigated the different mortality in locoregional progression compared to distant metastases (see for example [52, 53] Fig. 4, [54]). A hazard ratio of 0.35 is applied to the mortality rates generated from the 2LOS survival curves. The above assumptions were tested in a series of sensitivity analyses. Model assumptions for the underlying survival curves and transition parameters are listed in the appendix, Table 5.

Cost parameters reflect the perspective of German Statutory Health Insurance. Annual treatment costs for in Germany are mentioned within the dossiers submitted by manufacturers as part of the benefit assessment process required by the social security act (SGB V, section 35a). The costs related to adverse events from a German SHI perspective have been published [55]. Treatment and 1st line follow-up costs was based on the physicians‘fee schedule (EBM - *Einheitlicher Bewertungsmaßstab*) within the German Statutory Health Insurance Scheme [56]. Assumptions for the 1st line treatment mix were taken from an Italian source [57]. Best supportive care was assumed after the termination of the first line treatment using the results of [58] for NSCLC (Non-Small Cell Lung Cancer) in Germany. Palliative care was limited to the last 6 months before death. Costs for palliative care were based on the EBM rates. Tables 6, 7, 8, 9 and 10 in the appendix give a detailed overview of the cost assumptions made for the model.

Utility values were collected for the COMBI-AD trial [32] based on the US value set and EuroQoL EQ-5D-3L assessments made during the study using a Visual Analogue Scale (VAS) and evaluated by Gerbasi et.al [40] for the RFS, Local Recurrence, and Distant Metastasis Recurrence states. Gerbasi et.al. did not correct utility values in the case of adverse events, arguing that the impact of adverse events was already reflected by the COMBI-AD utility values. The base case assumptions concerning utilities are listed in Table 11 (appendix).

Age decrements for QALYs have not been implemented in the base case. Using EQ-5D assessments and a scoring algorithm based on US community preferences, Sullivan et.al [59]. developed a catalog reflecting the QALY decrements for chronic conditions. The marginal impact of ageing – separate from the effect of chronic conditions - was estimated at − 0.00029 QALY per life year. A model including age decrements for QALYs is part of the sensitivity analyses.

Sensitivity analyses included several assumptions concerning the model setup as well as the assumptions concerning transition probabilities in the Markov sub-model probabilities mentioned above. Sensitivity analyses tested the effect of changes in the discount rate, the model horizon and different assumptions concerning the fitting and extrapolation of the survival curves. The effect of shorter model cycles was tested in a simpler Markov model following the model structure presented by [40].

The programming of the model was conducted in Microsoft Excel version 2019 (Microsoft Inc., Redmond, WA) and in TreeAge Pro, Version 2021 R2.1 (TreeAge Software, LLC, Williamstown, MA). Statistical analyses were performed in R version 3.6.1 [60] with the use of R libraries „survival“, „flexsurve “and „flexsurvcure “for the fitting of parametric survival models, and „netmeta “for the estimation of indirect effects [61–64].

## Results

### Base case results

The model resulted in discounted lifetime costs of 155.1 T€ for the treatment option dabrafenib/trametinib. The mean remaining lifetime is 12.2 life years (LY), quality-adjusted 10.4 QALY. Observation as a model comparator resulted in life-time costs of 69.0 T€ and a remaining lifetime of 9.8 years (quality-adjusted 8.1 QALY). The incremental cost-effectiveness ratio is thus 37,800 € per QALY (Cost delta: 86.1 T€, QALY delta 2.28 years).

Table 1 shows the results for effectiveness and costs by treatment phase. Both effectiveness and cost results include the effect of adverse events.

**Table 1:** Base case results by health states

Base Case Results	Dabrafenib / Trametinib	Observation
Life Years, discounted (rate: 3%)		
Relapse Free Survival	10.13	6.77
Locoregional progression	0.75	0.80
Distant metastasis	1.32	2.23
**Total LY**	**12.20**	**9.80**
QALY, discounted (rate: 3%)		
Relapse Free Survival	8.79	5.88
Locoregional progression	0.66	0.70
Distant metastasis	0.89	1.51
**Total QALY**	**10.34**	**8.09**
Lifetime costs (T€, discounted by 3%)		
Treatment	112.1	–
Relapse Free Survival	3.4	2.5
Locoregional progression	0.2	0.23
Distant metastasis	39.4	66.3
**Total**	**155.1**	**69.0**

*LY* Life years, *QALY* quality adjusted life years

In terms of effectiveness measures, the model yields a gain of 2.40 life years due to the therapeutic alternative dabrafenib/trametinib (quality-adjusted: 2.28 life years). The additional lifetime gained is based on a longer period of stay in the health state “relapse free survival”. The costs of adjuvant treatment with dabrafenib / trametinib are partly compensated by lower costs for the treatment of distant metastases.

Wahler et.al [55]. analyzed the costs of adverse events, comparing the AE results published in studies Combi AD and Checkmate 238 from a German SHI perspective. Average per-treatment costs for adverse events amount to about 700 € for both dabrafenib/trametinib and nivolumab. On average and considering the entire patient cohort, the costs caused by AE play only a minor role. In a similar way, adverse events have a limited influence on the overall effectiveness. Beusterien et.al [65]. determined utility decrements for melanoma related adverse events in the UK and Australia using standard gamble. For dabrafenib/trametinib, the application of these utility decrements with a discount rate of 3% would result in a lifetime QALY reduction of − 0.072 years.

### Sensitivity analyses

The base case result was tested by several deterministic sensitivity analyses, covering general model parameters and model parameters involving a high degree of uncertainty. The results are shown in Table 2. The model reacts very sensitively to changes in the time horizon and discount rate. If the time horizon is shortened to 10 years, the QALY effectiveness measure is almost halved. The cost effect is strongest for the “placebo” strategy. The resulting ICER is 104 T€/QALY (cost delta: 90.5 T€, QALY delta 0.87 years). A discount rate of 0% leads to significantly higher costs in the “placebo” strategy. QALYs for the dabrafenib/trametinib and observation increase to 15.5 and 11.7 years, respectively, leading to an ICER of 21.7 T€/QALY (cost delta: 82.2 T€, QALY delta 3.79 years) for the comparison of dabrafenib/trametinib vs. observation. The base case result turned out to be stable when the underlying survival curves were uniformly shifted upwards resp. downwards. To test changes in the long-term projection, the convergence time from the mortality rates reported by Combi AD to general population mortality rates was extended from 8 to 10 years (base case) to 45 years, resulting in increased mortality rates for both dabrafenib/trametinib and observation for the remaining lifetime. This scenario led to slightly decreased discounted lifetime costs (dabrafenib/trametinib: − 1.5 T€, observation: − 2.2 T€) accompanied by decreased QALYs over the remaining lifetime (dabrafenib/ trametinib: − 0.67 years, observation: − 0.64 years) and an increased ICER of 38.5 T€/QALY.

**Table 2:** Deterministic sensitivity analyses

		Costs (T€)		Effectiveness (QALY)		ICER
		dabrafenib/ trametinib	observation	dabrafenib/ trametinib	observation	
Base Case results		155.1	69.0	10.41	8.14	37.800
Deterministic sensitivity analyses related to general model setup						
Costs and effectiveness undiscounted	Base case: 3%	165.3	83.0	15.48	11.69	21.677
Model horizon 10 years	Base case: Lifetime	149.1	58.6	5,74	4.87	104.092
Mortality rates converge to general population after 40 years	Base case: convergence after 8–10 years	149.2	59.9	11.62	9.06	34.865
Survival curves shifted downwards: hazard ratios for RFS/DMFS/OS 10% higher	Base case: HR = 1. published curves	157.1	71.6	9.82	7.48	37.751
Survival curves shifted upwards: hazard rates for RFS/DMFS/OS 10% lower	Base case: HR = 1. published curves	152.8	65.9	11.07	8.82	38.576
Deterministic sensitivity analyses related transition probabilities with high uncertainty						
Split locoregional vs. DM when progressing from relapse free state: 80% DM, 20% LR	Base case: 33% LR. 67% DM	164.0	78.1	10.35	8.07	37.658
Split locoregional vs. DM when progressing from relapse free state: 40% DM, 60% LR	Base case: 33% LR. 67% DM	139.0	51.5	10.56	8.27	38.111
Transition probability from locoregional state to DM not increased compared to progression from RFS state to DM	Base case: Transition prob. increased by hazard ratio 1.5	151.6	63.8	10.44	8.18	38.787
Mortality in locoregional state not decreased compared to distant metastasis state	Base case: Mortality decreased by hazard ratio 0.35	167.4	81.0	10.31	8,03	37.787

*QALY* Quality adjusted life years, *ICER* Incremental cost effectiveness ratio, *HR* Hazard ratio *RFS* Relapse-free survival, *DMFS* Distant metastasis free survival, *OS* Overall survival, *LR* locoregional state, *DM* distant metastasis state

The deterministic sensitivity analyses related to transition probabilities with uncertain values showed the insensitivity of the model result with respect to these parameters.

The ICER of dabrafenib/trametinib vs. observation was sensitive to the inclusion of the age decrement for QALYs developed by [59] and described in the methods section. The inclusion of a QALY decrement of 0.00029 per life year exceeding the age of 65 years has a small effect on the base case result. QALYs discounted by 3% during the remaining lifetime drop by 0.037 years (dabrafenib/trametinib) resp. 0.024 years (observation). The ICER of dabrafenib/trametinib vs. observation increases to 38.02 T€/QALY.

Shorter cycle lengths were simulated in a simplified alternative Markov model. Using cycle lengths of 1 month instead of 6 months (base case) led to lower costs (dabrafenib/trametinib: − 3.7%, observation: -5.2%) and less QALYs (dabrafenib/trametinib: − 2.3%, observation: − 4.5%). The ICER of dabrafenib/ trametinib vs. observation dropped to 34.4 T€/QALY (− 7.1%).

### Projection of nivolumab results

The technique that was applied to project the surrogate survival curves for nivolumab vs. observation to the modelling framework of the current study has been described above. Using the point estimates of the hazard ratios of dabrafenib/trametinib vs. nivolumab resulting from the indirect comparison (DMFS: 0.8827; OS: 0.8975; RFS: 0.9202), the model resulted in discounted lifetime costs of 118.1 T€. The mean remaining lifetime was 11.4 years (quality-adjusted 9.8 QALY). Table 3 shows the results for nivolumab broken down by health states. Compared with observation (see Table 10 for the results of the observation strategy), nivolumab achieves an incremental cost effectiveness ratio of 29.97 T€ per QALY (Cost delta: 49.1 T€, QALY delta 1.64 years).

**Table 3:** Base case results by health states, Nivolumab

Base Case Results	Nivolumab
Life Years, discounted (rate: 3%)	
Relapse Free Survival	9.48
Locoregional progression	0.76
Distant metastasis	1.21
**Total LY**	**11.44**
QALY, discounted (rate: 3%)	
Relapse Free Survival	8.23
Locoregional progression	0.66
Distant metastasis	0.82
**Total QALY**	**9.71**
Lifetime costs (T€, discounted, rate: 3%)	
Treatment	76.7
Relapse Free Survival	3.5
Locoregional progression	0.19
Distant metastasis	37.6
**Total**	**118.1**

*LY* Life-year, *QALY* Quality-adjusted life-years

As the estimated hazard ratios of nivolumab vs. dabrafenib/ trametinib as associated with large confidence intervals, the respective hazard ratios have been altered by +/− 20% as deterministic sensitivity analyses. Reduction by 20% in favor of dabrafenib/trametinib leads to hazard ratios of 0.72 (OS), 0,74 (RFS), and 0,71 (DMFS). Nivolumab lifetime costs increase to 123.1 T€. Nivolumab QALY drop to 8.40 years, which is close to the lifetime QALY associated with placebo (8.14 years). The ICER increases to 206.8 T€ per QALY (Cost delta: 54.1 T€, QALY delta 0.26 years). An increase by 20% in favor of nivolumab leads to hazard ratios of 1.08 (OS), 1.10 (RFS), and 1.06 (RFS). Nivolumab lifetime costs drop to 114.1 T€. Nivolumab QALY increase to 12.72 years, with is close to the lifetime QALY associated with dabrafenib/trametinib. The ICER drops to 16.3 T€ per QALY (Cost delta: 45.1 T€, QALY delta 2.78 years).

## Discussion

The aim of the present study is to evaluate the cost-effectiveness of options for the treatment of patients with stage III/IV melanoma after resection who have a BRAF V600 mutation. Within a German public sick funds setting the use of a combination of dabrafenib and trametinib was shown to be cost-effective applying internationally accepted thresholds. The base case resulted in higher costs (155.1 T€ vs. 69.0 T€), contrasted by a substantially longer remaining lifetime in years (12.2 LY vs. 9.8 LY) and an ICER of 37.8 T€ per QALY gained compared to observation as the alternative strategy. The result proved to be robust to changes in model parameters within the framework of deterministic sensitivity analyses. Scenarios reducing the model timeframe to 20 or 10 years resulted in substantially higher ICERs.

The projection of nivolumab results to the modelling framework of dabrafenib/trametinib resulting in lower costs compared to dabrafenib/trametinib associated with lower QALYs in the remaining lifetime and overall lower remaining lifetime. The resulting cost effectiveness ratio of nivolumab vs. observation comparable to the ICER of dabrafenib/trametinib vs. observation. Nevertheless, this result is subject to several uncertainties for methodological and statistical reasons.

Results for Germany are based on list prices of treatments of early 2020. Levels of possibly negotiated prices with single sick-funds are unknown to the public. Prices may change with the introduction of additional indications. Thus, the model results may undergo alterations with possible shifts in the price frame.

Similar considerations must be taken into account for the medical judgement. The follow-up data cuts in the study cohorts are still going on and each new evidence for the degree of improved long-term overall survival or relapse free survival will influence the model parameters. Thus, the analysis can only be a snapshot and the results may alter over time.

At this point of time only for the combination data for 60-month follow-up were available, with reported significant overall survival differences. This evidence is matched with assumptions for the other therapies.

During the approval process and the benefit assessment of immune-checkpoint inhibitors in the adjuvant therapy of stage III/IV melanoma, cost-effectiveness models for the various therapy options were developed. Models were created for the assessment procedures at the National Institute for Health and Care Excellence (NICE) [66, 67], the Canadian approval agency CADTH [68], the Irish National Centre for Pharmacoeconomics [69] and the Australian Benefit Assessment Commission PBAC [70], among others, whose results are only partially publicly available.

For nivolumab, due to immature data, no survival curves for overall survival were not available until late 2020. Different approaches to construct surrogates for the missing survival curve were presented [71, 72], which usually project results from study CA184–029 (ipilimumab vs. placebo) [21] to the relationship ipilimumab vs. nivolumab, which was investigated in study Checkmate-238 [33]. Batteson et.al [71]. presented an alternative surrogate based on systematic research of available literature. On this basis, a series of country-specific cost-effectiveness calculations for the relationship nivolumab vs. observation were presented as congress papers (for Spain [73], Greece [74], the National Health Service (UK) [72], Switzerland [75]). Also as congress papers, a comparison of the combination therapy dabrafenib/trametinib vs. observation for Canada [76] with observation and a comparison of dabrafenib/trametinib with pembrolizumab for Brazil [77] were presented.

Due to country-specific differences and differences in model design and assumptions, there are strong fluctuations in the model results. The result of the present study is roughly comparable to Gerbasi’s base case ICER ($34,689) [40] and the results for Canada (base case ICER: CAN$ 28,865) [76] comparing dabrafenib/trametinib with observation. For the comparison of pembrolizumab vs. observation, [41] showed an ICER of $15,009 per QALY.

The lower ICER values shown by [40] originate from a different modelling of the locoregional and distant metastasis progression phases, resulting in an increased overall survival time for the dabrafenib/trametinib treatment branch compared to observation (13.0 vs. 10.6 life years; 11.0 vs. 8.8 QALYs). The increased survival time shown by Gerbasi et.al [40]. is also caused by the different modelling technique. While Gerbasi et.al. model death as state transitions in a Markov model, the present study employs Partitioned SA, thus adopting mortality rates as predetermined by the published survival curves.

The modelling framework of the present study was also applied to compare nivolumab with observation. A complete set of 4-year efficacy results for the comparison of nivolumab versus ipilimumab was published in 2020 [37]. These results were used for two subsequent indirect comparisons, nivolumab vs. observation with ipilimumab as a bridge comparator and dabrafenib/trametinib vs. nivolumab with observation as a bridge comparator. The resulting ICER of nivolumab compared with observation was 30.0 T€/QALY. Due to the lack of availability of patient level data, the potential bias caused by the imbalance of patient characteristics, as shown in Table 4, could not be assessed by sensitivity analyses.

Ntais et.al [72]. showed an ICER value of £ 18,018/QALY for the relationship nivolumab vs. Observation as result of a Markov model from the perspective of the National Health Service in the UK. The National Institute for Health and Care Excellence (NICE) Evidence Review Group noted that alterations of certain model assumptions (like the cost assumptions for 1st and 2nd line treatment after the adjuvant phase) would cause the ICER to be slightly higher [67].

The aspect of adverse events during the one-year treatment period was found to be of economically minor relevance in comparison with the valuated survival gains. Nevertheless, the side effect structure of both treatments is rather different. For the combination more, but minor events were reported. A rigorous analysis of the economic impact resulted with both therapies on the same level. There is no data available for QALY losses due to long-term disabling side effects, mostly with nivolumab.

Given those factors the result of the cost-effectiveness analysis from the perspective of the German system indicates that the treatment decision for dabrafenib/trametinib or nivolumab with equal cost effectiveness will remain based on individual clinical parameters, evidence about long-term overall survival and avoidance of severe adverse events.

### Limitations

The model presented here consists of a mixture of a Partitioned SA approach, which takes into account the survival curves obtained in the Combi-AD trial, with a Markov state transition model reflecting the proportions of patients in the progression stages. Advantages and disadvantages of the two methods are discussed in the literature [71, 78]. The use of Partitioned SA as an outer model has the advantage that the empirically determined survival curves of the first 60 months are not distorted by further model assumptions. In contrast, the proportions of patients in the progression stages could not be represented by a Partitioned SA model, because survival curves for the corresponding state transitions (e.g., locoregional to distant metastasis) have not been published. These model states are necessary because they differ substantially in terms of cost and utility values. A limitation of the method is that the parameterization of the (inner) Markov model is based on assumptions that are essentially based on the model of Gerbasi et.al [40]., who in turn evaluated patient level data. The effect of these assumptions was tested in sensitivity analyses. Another disadvantage is the fact that mortality outcomes are different in both parts of the model. This conflict was resolved by assigning priority to the mortality outcome of the Partitioned SA part of the model.

The model presented here for the comparison of dabrafenib/trametinib uses study results that report OS and RFS survival for a follow-up period of 60 months maximum [32, 46]. As the time horizon of the model covers the remaining lifetime of the patients, assumptions were necessary for the long-term shape of the survival curve. Without empirical evidence, an approximation of mortality rates to the mortality of the general population in Germany (destatis) seemed to be reasonable. Sensitivity analyses of the long-term curve carried out by Gerbasi et.al [40]. showed that their model result was highly dependent on the assumptions.

The cycle length of 6 months assumed in the base case may be too long to estimate short-term effects after the start of treatment with sufficient accuracy. Other authors used shorter model cycles [41, 72]. However, a sensitivity analysis revealed a relatively small influence of cycle length on the model result.

The quality-of-life assumptions for the model originate from the EQ-5D values gathered during the underlying study Combi-AD [40] using a US value set and a visual analogue scale (VAS). A validation of EQ-5D data for Germany [79] concluded that EQ-5D values are likely to reflect cultural differences between countries, especially if collected by time trade-off methods. The present study assumes the transferability of the Combi-AD utility values to Germany.

The main source for the cost assumptions is the current EBM catalogue of the National Association of Statutory Health Insurance Physicians (KBV) [56]. Although the therapy alternatives for advanced melanoma (1st line, 2nd line) are based on the guidelines for Germany [80], costs of therapy alternatives were taken from an Italian source [57]. The cost assumption for “best supportive care” has been adopted from a study for non-small cell lung cancer [58]. It is likely that the costs of best supportive care for advanced melanoma differ from this assumption. For 2nd line therapies, “best supportive care” was assumed to be the only therapeutic alternative due to the lack of available information.

The present study attempts to project the comparison of nivolumab with observation into the model framework of the comparison of dabrafenib/trametinib with observation. To this purpose, two subsequent indirect comparisons (nivolumab vs. observation using ipilimumab as bridge comparator, dabrafenib/trametinib vs. nivolumab using observation as bridge comparator) were performed. The current approach did not take the different definition of target populations (stages IIIa-IIIb for dabrafenib/ trametinib, stages IIIb-IIIc, IV for nivolumab) into account. Furthermore, there are notable differences the population characteristics of the studies involved. Out of these reasons, the resulting comparison of nivolumab vs. observation within the model framework of this study is subject to multiple uncertainties. Patient-level data are needed to assess and potentially adjust for the impact of the observed lack of balance in patient characteristics.

## Data Availability

All data generated or analyzed for the economic model are included in this published article.

## Appendix

### Model parameters

**Table 4:** Population characteristics of the involved studies

Study	Combi-AD [32]		EORTC-19071 [18]		Checkmate-238 [33]	
Branch	Dab + Tram	Placebo	Ipilimumab	Placebo	Nivolumab	Ipilimumab
Number of Patients	438	432	475	476	453	453
Gender (Male)	44.5	44.7	62.3	61.6	57.0	59.4
Age: Median, Range	50 (18–89)	51 (20–85)	51 (20–84)	52 (18–78)	56 (19–83)	54 (18–86)
Proportion < 65 years	Not reported		82.9	81.7	73.5	74.8
**Disease Stage**						
IIIa (%)	18.9	16.4	20.6	20.6	–	–
IIIb (%)	38.6	43.3	38.3	38.2	36.0	32.7
IIIc (%)	41.3	38.4	25.7	25.4	45.0	48.1
III (Not Specified, %)	1.1	1.9				
IV (%)			15.4	15.8	18.1	19.2
Not Reported			–	–	1.0	–
**Stage III lymph node involvement**						
Microscopic (%)	34.7	36.3	44.2	40.5	33.9	36.6
Macroscopic (%)	36.1	37.3	55.8	49.5	59.3	58.5
Not Reported	29.2	26.4	–	–	6.8	4.9

*Dab+Tram* dabrafenib/trametinib combination therapy. Sum of percentages may deviate from 100% due to rounding errors

**Table 5:** Partitioned survival analysis setup and transition probabilities

Partitioned Survival Analysis	Branch	Sources
Overall Survival (OS)	Dab+Tram Placebo	Combi AD (54 months), Long (2017, Fig. 1B) [32]
Distant Metastasis Free Survival (DMFS)	Dab+Tram Placebo	Combi AD (60 Months), Hauschild (2018, Fig. 2B) [46]
Relapse Free Survival (RFS)	Dab+Tram Placebo	Combi AD (60 Months), Hauschild (2018, Fig. 2A) [46]
Overall Survival, Relapse Free Survival (RFS), and Distant Metastasis Free Survival (DMFS)	Nivolumab	Indirect comparisons using Hauschild (Dab+Tram vs. Placebo) [46], Eggermont (Ipilimumab vs. Placebo) [21] and Ascierto (Nivolumab vs. Ipilimumab) [37]]
**Markov sub-model for disease progression**		**Sub-model structure based Gerbasi et.al. (2019)** [40]
Transition RFS - > Death	All branches	General mortality for the German population (starting age 50 years) [41]
Transition RFS - > Progression	All branches	Assumption RFS- > Locoregional 33%, RFS- > Distant metastases 67% (based on [46], Table 1)
Transition locoregional progression - > Distant metastases 1st line treatment	All branches	Based on DMFS associated to the respective treatment branch. Hazard ratio increased by a factor of 1.5 (assumption) (similar approach as in [40]). Level of increase is lower compared to [40]
Transition Locoregional progression - > Death	All branches	Hazard ratio for distant metastases, 2nd Line - > Death (2LOS) in [40], Fig. 2C. Hazard ratio decreased by factor 0.35 (assumption)
Transition distant metastases 1st line treatment - > 2nd line treatment	All branches	Hazard ratio for Distant metastases, 1st line - > 2nd line. Source: [40], Fig. 2B.
Transition distant metastases 1st line treatment - > Death	All branches	Hazard ratio for distant metastases, 2nd Line - > Death (2LOS). Source: [40], Fig. 2C
Transition distant metastases 2nd line treatment - > Death	All branches	Hazard ratio for distant metastases, 2nd Line - > Death (2LOS). Source: [40], Fig. 2C

*Dab+Tram* dabrafenib/trametinib combination therapy, *OS* Overall survival, *DMFS* Distant-metastasis free survival, *RFS* Relapse-free survival, *2LOS*: 2nd line overall survival

**Table 6:** Cost parameters: medication and adverse events

	Cost (€)	Share of patients	Sources
**Dabrafenib/trametinib**, annual costs, including application	114,411 €		Source: manufacturer dossier [81], price adjustment 2019/2020
**Nivolumab**, annual costs, including application	78,460 €		Source: manufacturer dossier [82]
**Adverse events**	**€ per patient**		
AE degree 1–2, dabrafenib/trametinib	227.37 €	55.6%	
AE degree 3–4, dabrafenib/trametinib	113.26 €	41.4%	
AE degree 1–2, observation	96.45 €	73.8%	
AE degree 3–4, observation	14.86 €	14.1%	
AE degree 1–2, nivolumab	231.92 €	71.3%	
AE degree 3–4, nivolumab	179.42 €	25.4%	

*AE* Adverse events

**Table 7:** Cost parameters: BRAF testing and routine screening

	Cost (€)	Sources
BRAF test (per test) -	120.00 €	Source: German Society for Hematology and Oncology (www.dgho.de [83])
BRAF-Test (per positive test)	265.00 €	Source: manufacturer dossier [82]
Follow-up costs 1 (flat fee for dermatologists), per quarter	17.16 €	EBM 10212 + 10,220, 15,24 € + 1,92 €
Follow-up costs 2 (Sonography, lymph nodes), pro examination	8.01 €	EBM 33080
Follow-up costs 3 (Biomarker S100beta), per examination	22.70 €	EBM 12210 basis fee and consultation fee; EBM 32405: S100 Biomarker
Follow-up costs 4 (imaging), per examination	408.98 €	EBM codes 34320, 34321, 34311, 34342, 34350, 34351
Follow-up costs 5 (full body scan)	12.23 €	EBM 27310
Number of screenings according to German guidelines ([80], section 8: Follow-up): Years 1–3: 4x Follow-up 1, 4x Follow-up 2, 4x Follow-up 3, 2x Follow-up 4 Years 4–5: 4x Follow-up 1, 4x Follow-up 2, 4x Follow-up 3 Years 6–10: 2x Follow-up 1 After year 10: 1x Follow-up 5		

*EBM* Einheitlicher Bewertungsmaßstab [56]

**Table 8:** Cost parameters: treatment, locoregional progression

	Value	Sources
Inpatient treatment (share of patients)	10%	Assumption
Inpatient treatment, cost per case	2263.78 €	GDRG-Grouper [84]: ICD10 C43.9, OPS 5–894, 5–895
Outpatient treatment, cost per case	815.34 €	EBM 31164 272.30 €| EBM 31505 111.58 €| EBM 31610 31.60 €| EBM 31611 25.22 €| EBM 31824 197.84 €| EBM 34450 131.28 €| EBM 34220 10.28 €| EBM 33042 16.99 €| EBM 33081 7.25 €| EBM 32392 9.20 €
Locoregional progression with adverse events: share of cases	5%	Assumption: patients with AE are re-treated with the same costs

*ICD* International Statistical Classification of Diseases and Related Health Problems, *OPS* Operationen- und Prozedurenschlüssel (German operation and procedure code), *GDRG* German Diagnosis Related Groups, *AE* Adverse events, *EBM* Einheitlicher Bewertungsmaßstab [56]

**Table 9:** Cost parameters: distant metastases (diagnostics)

	Value	Sources
All diagnostics	875.64 €	Deutsche Krebsgesellschaft: Guidelines 2018 [80]
MRT: head	131.28 €	EBM 34421 MRT basal skull
PET/CT: full body	611.80 €	EBM 34701 including CT diagnostics
Sonography: abdomen	16.99 €	EBM 33042
Sonography: lymph nodes	8.01 €	EBM 33080
Scintigraphy: skeleton	70.11 €	EBM 17311 Full body scintigraphic examination
Tumor marker S100B	22.80 €	EBM 32405
Tumor marker LDH	0.25 €	EBM 32075
Consulting fees radiology, laboratory	14.40 €	EBM 24212, 12,210

*EBM* Einheitlicher Bewertungsmaßstab [56]

**Table 10:** Cost parameters: distant metastases (treatment)

	Annual costs	Share of patients	Duration	
**Advanced melanoma treatment: immuno-suppressant drugs**	**108,192 €**	**85%**	1 year	
Dabrafenib + Trametinib	114,411 €	12%		Quelle: Dossier [81], price adjustment 2019/20
Ipilimumab	91,788 €	23%		Dossier Ipilimumab, advanced melanoma [85]
Nivolumab	78,460 €	6%		Dossier Nivolumab [82]
Pembrolizumab	130,858 €	13%		Dossier Pembrolizumab, Advanced Melanoma [86] Assumption: 60% men, 40% women
Vemurafenib	93,108 €	1%		Dossier Cobimetinmib 2015, advanced melanoma [87]; Vemurafenib as comparator
Nivolumab + Ipilimumab	139,958 €	8%		Dossier Nivolumab+Ipilimumab, advanced melanoma [88]
Vemurafenib + Cobimetinib	90,637 €	3%		Dossier Vemurafenib+Cobimetinib, advanced melanoma [87]
**Chemotherapy**	**9194 €**	**5%**	unlimited	Dacarbazin treatment (used in dossier [89] as comparator for Nivolumab+Ipilimumab, mean of therapy frequencies (1 × 17, 5 × 17)
**Surgery**	**3000 €**	**20%**		Assumption (standard inpatient flat rate per case in Germany)
**Radiotherapy**	**4776 €**	**5%**	1 year	KBV statistic 2012, following [90], S, 9
**“Best supportive care”**	**17,531 €**	**5%**	unlimited	Schmidt/Lipp/Drechsler, ISPOR 2014, poster PCN86 [58]
Frequency of immunosuppressant therapy: Gerbasi [40] Frequency of therapies in general: assumption following Maio et.al, [57]				
2nd line treatment	17,531 €			Best supportive care, see above [58]

*KBV* Kassenärztliche Bundesvereinigung (National Association of Statutory Health Insurance Physicians)

**Table 11:** Utility parameters

Health state	QALY	Source / Comment
Progression free survival (RFS)	0.875 (Treatment independent)	Gerbasi et.al [40]., Table 3 based on a post-hoc analysis of individual patient EQ-5D data in study CombiAD [32]
Progression locoregional	0.875	Based on Gerbasi et.al [40]., Table 3
Progression distant metastases 1st line treatment	0.724	Based on Gerbasi et.al [40]., Table 3
Progression distant metastasis, 2nd line treatment	0.653	Based on Gerbasi et.al [40]., Table 3

*RFS* Relapse-free survival

## Abbreviations

Dab+Tram: Dabrafenib/trametinib combination therapy
TafMek: Tafinlar®/Mekinist® (dabrafenib/trametinib combination therapy)
T€: Thousand Euros
QALY: Quality-adjusted life-year
VAS: Visual Analogue Scale
LY: Life years
ICER: Incremental cost-effectiveness ratio
DMFS: Distant metastases free survival
RFS: Relapse-free survival
OS: Overall survival
1PFLS: 1st line progression free survival
2LOS: 2nd line overall survival
LR: Locoregional state
DM: Distant metastasis state
Partitioned SA: Partitioned survival analysis
CI: Confidence interval
HR: Hazard ratio
SHI: Statutory health insurance
SGB: Sozialgesetzbuch (Social Security Act)
EBM: Einheitlicher Bewertungsmaßstab (uniform assessment standard – physicians’ fee schedule)
KBV: Kassenärztliche Bundesvereinigung (National Association of Statutory Health Insurance Physicians)
NSCLC: Non-small cell lung cancer
